# Supplementary figures and images for: Systemic Biomarkers and Unique Pathways in Different Phenotypes of Heart Failure with Preserved Ejection Fraction
Source: Biomolecules. 2022 Oct 4;12(10):1419. doi: 10.3390/biom12101419 (PMC9599828; doi:10.3390/biom12101419)

# Regulation of IGF transport and uptake by IGFBP

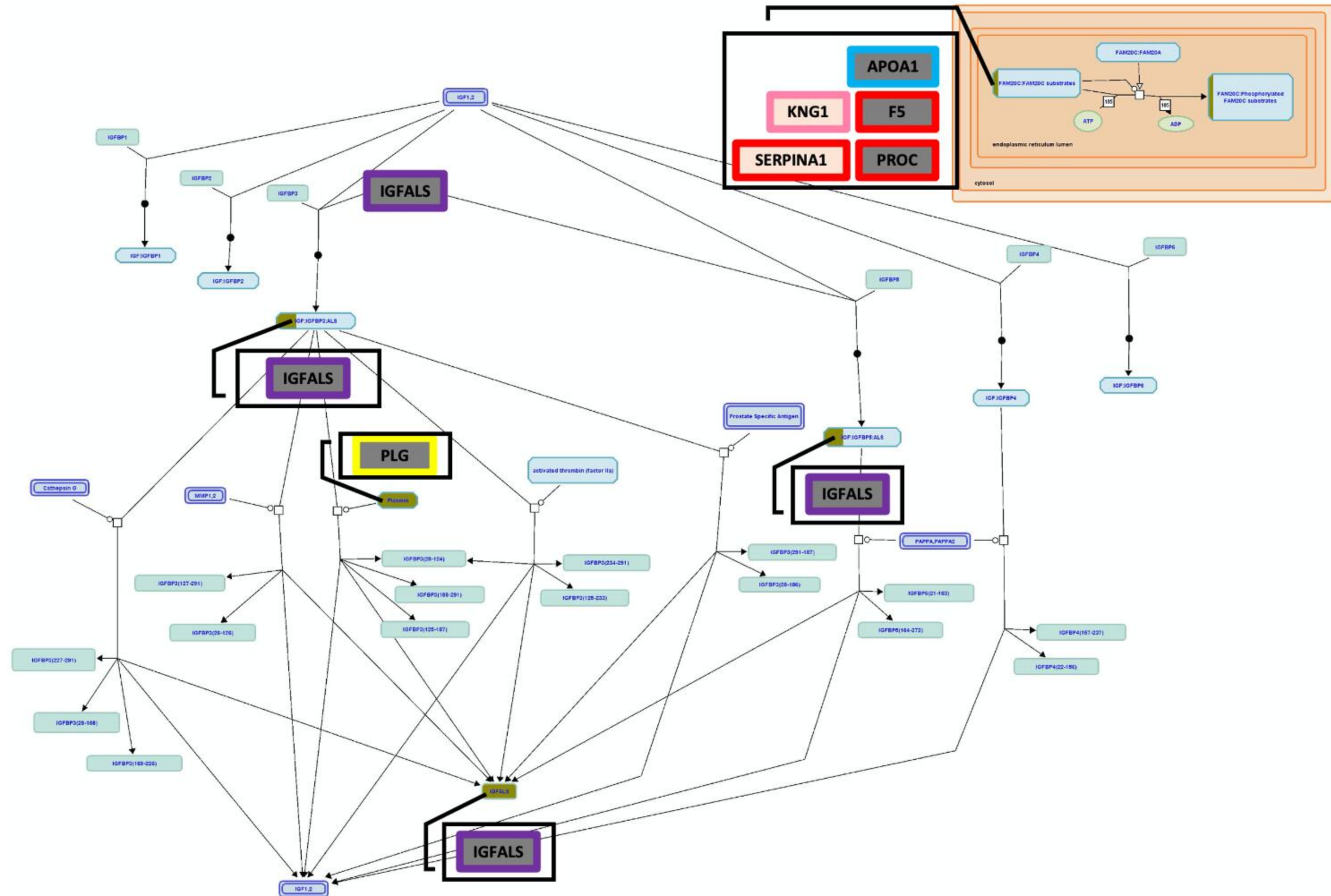

Supplement: Supplementary file 1 [file biomolecules-12-01419-s001.zip › Supplementary Figure S1.pdf]
